# A longitudinal study of the association of epidural anesthesia and low-dose synthetic oxytocin regimens with breast milk supply and breastfeeding rates

**DOI:** 10.1038/s41598-023-48584-6

**Published:** 2023-11-30

**Authors:** Kaori Takahata, Shigeko Horiuchi, Ai Miyauchi, Yuriko Tadokoro, Takuya Shuo

**Affiliations:** 1 Department of Nursing, Shonan Kamakura University of Medical Sciences, Yamasaki 1195-3, Kamakura, Kanagawa 247-0066 Japan; 2 Department of Midwifery, Graduate School of Nursing Science, St. Luke’s International University, Tokyo, Japan; 3 Department of Maternal Health, Japanese Red Cross College of Nursing, Tokyo, Japan; 4 Department of Chiba Faculty of Nursing, Tokyo Healthcare University, Chiba, Japan; 5 Faculty of Health and Medical Sciences, Hokuriku University, Ishikawa, Japan

**Keywords:** Biomarkers, Health occupations, Medical research, Risk factors

## Abstract

Breastfeeding is known to improve maternal and child health. However, epidural anesthesia (EDA) and synthetic oxytocin (synOT) are suggested to have negative effects on breastfeeding. In this study, we aimed to determine the effects of intrapartum synOT and EDA on breast milk supply, breastfeeding rates, and maternal salivary oxytocin levels. Women were recruited during pregnancy or after birth at a single hospital. Data were collected at 3 days postpartum (T1), 1 month postpartum (T2), and 4 months postpartum (T3) on 83 low-risk primiparous women who planned to breastfeed for at least 12 weeks postpartum to avoid dropouts from early discontinuance of breastfeeding. Women with cesarean section, twin pregnancy, premature neonates, and an Apgar score of < 7 at 5 min were excluded. Participants recorded their 24-h milk supply by test weights at 3 days and 1 month postpartum. Additionally, they filled out questionnaires assessing their breastfeeding level and lactogenesis stage II initiation. Salivary oxytocin levels were obtained at 3 days postpartum. Women who delivered using EDA had lower salivary oxytocin levels (*P* = .055,* d* = .442), breast milk supply in early postpartum (*P* = .025, *d* = .520) and at 1 month postpartum (*P* = .036, *d* = .483), and breastfeeding rates at 4 months postpartum (*P* = .037, *V* = .236) than women who did not deliver using EDA. There was no association between breastfeeding and the use of intrapartum synOT. In conclusion, this study showed that women who delivered using EDA had lower breast milk supply in the early postpartum period and breastfeeding rates at 4 months postpartum. It also revealed that using synOT at low doses during labor did not affect breastfeeding. Thus, women who deliver using EDA need support for increased breast milk supply in the early postpartum period.

Trial registration**:** UMIN000037783 (Clinical Trials Registry of University Hospital Information Network).

## Introduction

Synthetic oxytocin (synOT) and epidural anesthesia (EDA) are common medical interventions that promote labor and relieve pain. In particular, synOT has been assessed as medically applicable and recommended for elective use, and its appropriate administration and dosage have also been explored^1–4^. EDA is the preferred method of relieving pain associated with childbirth. However, one of the long-term effects of medical interventions that may persist after delivery is their negative effects on breastfeeding^5,6^. Intrapartum synOT has been shown to decrease breastfeeding rates and shorten breastfeeding duration^7^. The higher the dose of synOT, the lower the breastfeeding rate at 2 months postpartum^8^. An integrated review of 34 articles found that the use of synOT was associated with delayed breastfeeding initiation, shorter breastfeeding duration, and decreased infant feeding behavior in 50% of studies^6^. Notably, the use of EDA has also been found to be associated with delayed lactation initiation and shorter breastfeeding duration^9,10^.

Endogenous oxytocin (OT), whose secretion increases during pregnancy and parturition and plays an essential role in increased milk supply, has been reported to be affected by synOT and EDA. When synOT and EDA are combined, plasma OT decreases on day 2 postpartum^11^. The use of synOT during parturition has also been found to affect maternal endogenous OT even at 2 months postpartum^8,12^. Furthermore, as synOT decreases OT receptor mRNA levels in the human uterine myometrium by 60–300-fold^13^, it is highly likely that OT receptor downregulation also occurs in the breast. Although some reports in the literature suggest that synOT does not cross the placenta, dose-dependent effects, maternal weight changes, and other alterations have been pointed out^14–16^. Moreover, reports of substantial weakening of primitive reflexes in neonates^17^ indicate that synOT may affect the fetal brain.

SynOT and EDA have been suggested to affect newborns’ primitive reflexes and mothers’ milk secretion through their effects on maternal endogenous OT secretion and receptors. However, the effects of synOT and EDA may have been underestimated to date as previous studies have assessed breastfeeding mainly by surveys through phone calls to mothers. Although several studies have examined the association between salivary OT and milk supply, to our knowledge, there are still no studies that have investigated the impact of medical interventions (e.g., use of synOT and EDA) on milk supply^18–20^. A thorough understanding of the early postpartum milk supply of women who have received synOT and EDA and the long-term effects of these medical interventions have important implications for perinatal care. Thus, this study aimed to determine the effects of intrapartum synOT and EDA on breast milk supply, breastfeeding rates, and maternal salivary OT levels.

## Methods

### Study design and participants

This study used a cohort design. Data were collected from September 2019 to April 2021 in a single maternity hospital in an urban area in Tokyo, Japan. However, data collection was temporarily interrupted for 4 months from April 2020 to July 2020 because of COVID-19 following the Japanese government guidelines.

Women were recruited during pregnancy or within 48 h of giving birth in the maternity hospital. The eligibility criteria of the participants considered milk secretion, preference for breastfeeding, and effects on OT secretion and receptors as follows: (1) between 20 and 43 years of age, (2) having singleton and spontaneous delivery as low-risk pregnancy, (3) planning to continue breastfeeding for at least 12 weeks postpartum (including mixed feeding), and (4) Asian who can read and write Japanese.

For the exclusion criteria, women were excluded from the study if they have (1) ongoing medications, (2) medical or pregnancy complications, (3) a history of breast surgery, (4) current history of mental illness, epilepsy, or cancer, (5) cesarean section, (6) babies whose birth weight was < 2500 g, (7) preterm birth, (8) babies whose Apgar score was < 7 at 5 min, or (9) other conditions causing difficulty in breastfeeding. Additional exclusion criteria were based on the salivary OT level assay, including (10) taking or inhaling steroids, (11) with HIV, HCV, or HBV, and (12) dental illness.

### Procedures

We conducted longitudinal data collection at 3 days postpartum (T1), 1 month postpartum (T2), and 4 months postpartum (T3). In Japan, women usually stay in the hospital until the fifth day postpartum and receive breastfeeding counselling. Therefore, at 3 days postpartum in the maternity hospital, the researchers (KT and AM) visited a patient’s private room and collected a saliva sample after obtaining written consent for the study (T1). The breast was then observed and reviewed with the patient for breast fullness status on the day, and a record form on breast fullness was filled out for the previous 3 days. A high-accuracy baby scale was placed in a safe location in the room. The test weight was explained to the participants with practical examples. The *milk supply for 24 h* was assessed as the total amount of test weights to assess breastfeeding and milk supply after pumping. Questionnaires and recording forms were collected by posting them in a box located in the hospital or returning the enclosed envelope.

For data collection at 1 month postpartum (T2), subjects decided the participation date and recorded their 24-h breastfeeding supply using a pre-maintained, high-accuracy baby scale that was delivered to their homes. Questionnaires and recording forms were collected using the enclosed envelope. For data collection at 4 months postpartum (T3), the women were asked to fill out a questionnaire collected using an enclosed envelope. Gratuities to the participants were paid by bank transfer according to the number of times they participated.

### Measures

The participants provided demographic information about their parity, age, marital status, and years of education. Additionally, synOT dose, EDA dose, and other data were collected from hospital charts.

#### Breast milk supply and breastfeeding

The measurements of the 24-h milk supply by test weights were obtained at T1 and T2. As a surrogate indicator of milk supply in lactation, the infant intake test weight has been implemented^21,22^. In accordance with the recommended methods, standardized guidance was provided to improve the accuracy of the test weights. Baby scales (TANITA Corp., High Precision Baby Scale with official approval, BD-815) were sent to participants’ homes, each time calibrated by the rental company. We standardized the guidance as follows: (1) if necessary, *change* the diaper before weighing; (2) *weigh* the infant on a baby scale while wearing a diaper and clothing; (3) after the feeding, *weigh* the infant on a baby scale without changing the diaper; (4) *feed* pumped breast milk *or* artificial milk after weighing, if necessary.

Data were collected at a single facility. Therefore, all participants received essentially the same breastfeeding counselling and care during pregnancy and after labor. In the facility where data were gathered, mothers were educated about the benefits of breastfeeding during pregnancy, and their preferences for breastfeeding were confirmed up to delivery. After delivery, a 5-day postpartum hospital stay allowed the mothers to receive proper care and counselling. At 2 weeks and 1 month postpartum, the mothers had an opportunity to visit the facility to check their child’s nutritional condition. In addition, local medical staff would visit the mothers’ homes to check their child’s nutritional condition free of charge. Mothers could make their own appointments to consult with a local midwife at their own expense.

The initiation date of lactogenesis stage II was determined using the *scale of breast fullness* developed by Dewey et al.^23^. A daily 5-Likert scale response for breast fullness was given, and the approximate time when “obviously full” was first reached was noted. In this study at day 3 postpartum, the breast condition was checked by a midwife, and breast fullness was agreed upon with the participant. Participants then kept a record of their breast status until day 9 postpartum.

The Level of Breastfeeding scale was used to assess the proportion of breastfeeding in the total infant diet^24^ at T2 and T3. This scale classifies breastfeeding into 3 categories: Full, Partial, and Token. *Full breastfeeding* is categorized as Exclusive (no oral intake other than breast milk) and Almost Exclusive (vitamins, minerals, water, etc. can be given). *Partial breastfeeding* is classified as High, Medium, or Low, depending on the percentage of breastfeeding. *Token breastfeeding* is feeding the child for nursing no more than 15 min at a time and no more than 2–3 times per day with little or no nutritional impact. In the present study, the Level of Breastfeeding was categorized as High breastfeeding (≥ 80% of the total infant diet) or Low breastfeeding (< 80% of the total infant diet).

#### Saliva collection and assay

As in our previous studies, strict environmental controls were implemented^25,26^. Saliva collection was standardized between 12:00 and 5:00 pm. Caffeine and alcohol should not be consumed on the day of saliva collection, specifically 12 h before the start of saliva collection^27^. Eating and brushing teeth should be completed at least 60 min before saliva collection^27^ Lipstick should be wiped off before saliva collection^28^. Saliva samples were collected at least 30 min after the end of breastfeeding. The newborn was attended to in the neonatal room by a midwife. Only 1 participant and the researcher were allowed in the private room. Pictures of dried plums and lemons were placed on an overhead table to stimulate salivation. Two researchers (KT and AM) collected data. Cell phone use, drinking water, and other nonresearcher-directed activities were restricted during saliva collection. The participants rinsed their mouths at the washbasin and drank 100 ml of water. They initially viewed a video of experimental precautions and instructions on how to collect saliva. Then, they watched a DVD video (i.e., Relaxing River Sounds) with headphones 10 min before saliva collection to standardize their conditions and minimize the effect on oxytocin.

Saliva was collected by the drooling method using a straw. Women waited for 1 min to pool the saliva spontaneously. Then, the saliva was passed through the tube using a straw for 1 min, followed by a 30-s rest. This was repeated at least 3 times to ensure a minimum saliva volume of 1.5 ml. The saliva samples were immediately stored in a portable freezer at − 80 °C. OT level was assayed using the method of Carter et al.^29^. The protease inhibitor aprotinin (500 KIU/mL) was added to inhibit the metabolic breakdown of the peptide after thawing the saliva^30^. OT assays were performed for purification by solid-phase extraction, and OT concentration was determined by enzyme-linked immunosorbent assay (Enzo Life Sciences, Farmingdale, NY, USA). All samples were run in duplicate assay.

#### Medical interventions

In Japan, a low-dose regimen of synOT has been used in accordance with the *guideline for obstetrical practice in Japan 2020*. The use of synOT was performed by midwives as indicated by the responsible obstetrician. An infusion of 5 IU of synOT was added to a 500 mL of saline solution [10 milli-unit/minute (mU/min)]. Administration of 1–2 mU/min was initiated, and the dose rate was increased by 1–2 mU/min every 30 min until effective contractions were achieved, up to a maximum of 20 mU/min. In some cases, synOT was used for several days after hospital admission until delivery. All mothers received OT after their delivery to prevent bleeding in the third stage of labor. The synOT group was classified according to whether synOT was administered during parturition. Any synOT administered for inducing labor was also combined.

Women who wished to have an EDA received a predelivery explanation from the anesthetist. EDA could be performed whenever a woman requested it, and an anesthetist or a perianesthesia nurse administered it. EDA consists of the local anesthetic ropivacaine in combination with fentanyl; mepivacaine was used as a test dose.

### Statistical analysis

In the absence of similar previous studies, there is a lack of data available for estimating an appropriate sample size. Therefore, for this study, the maximum number of cases collected during the study period was analyzed. A post hoc analysis of the data examining early postpartum milk supply in the *with and without EDA groups* showed an effect size of 0.52 and an alpha error of 0.05, with Power 0.62.

For demographic data and variables, these were compared across all data, with and without EDA, and with and without synOT, with basic statistics calculated. For breast milk supply, *t*-tests were performed, and Cohen’s d was reported as effect sizes. Intrapartum synOT results were presented as medians and interquartile ranges (IQR; 25th–75th percentile). Additionally, Fisher’s exact test was performed for high breastfeeding, and Cramer’s V was reported as the effect size.

A midwife checked the lactation records for feeding frequency, milk expression, and artificial milk supplements, and repeatedly checked for unnatural data. If more than half of the lactation records were missing, they were excluded from the sample as insufficient data.

All data were analyzed using SPSS version 28.0 (SPSS Inc., Chicago, IL, USA). A two-tailed *P* < 0.05 was considered to indicate a statistically significant difference.

### Ethical approval and consent to participate

This study was performed in accordance with the Japanese Ethical Guidelines for Medical and Health Research Involving Human Subjects, and an approval from St. Luke’s International University Research Ethics Review Committee (18-A077, January 17, 2019). This study was registered the University Hospital Medical Information Network Clinical Trials Registry (UMIN000037783). All participants provided written informed consent for the publication of the results of this study. The reported content followed the STROBE checklist for cohort studies.

## Results

**Figure 1:**
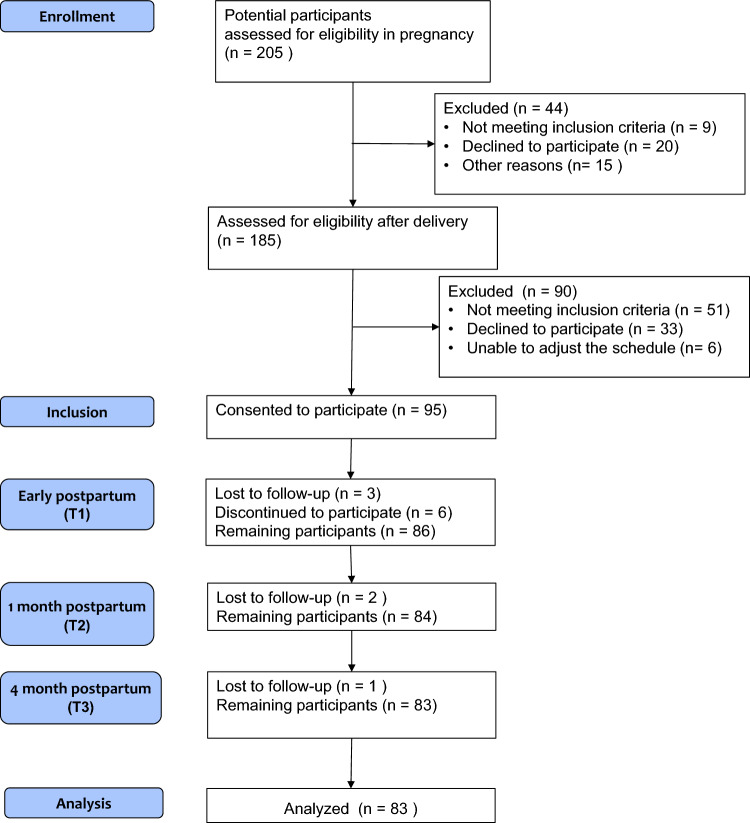
Flow diagram describing the eligibility and analysis of women.

Figure 1 shows a flow diagram describing the eligibility and analysis of the participants. After enrollment and exclusion, 95 participants consented to be included and had their saliva samples taken; eventually, 86 participants provided early postpartum lactation records at T1, then 84 participated in the first postpartum month follow-up (T2), and finally 83 participated in the fourth postpartum month follow-up (T3).

Table 1 shows the sociodemographic characteristics of the participants. The participants had a mean age of 33.5 years (SD 3.7), gestational age of 39.6 weeks (SD 1.0), were non-obese, mostly married (98.8%), had jobs (86.8%), and mostly had a university education (98.8%). Some participants had histories of polycystic ovarian syndrome (9.6%), and more than half decided to breastfeed before 13 weeks during pregnancy (57.8%). Mothers with fetal anomalies were not included in this study. Blood loss during parturition was slightly higher at 574 g (SD 300), and Hb was slightly lower at 9.8 mg/dL (SD 1.3), significantly higher in the synOT group than in the non-synOT group. There were no significant differences between with and without EDA or with and without synOT. Most performed skin-to-skin contact (SSC) within the first hour of delivery (91.6%), and SSC within 1 h after childbirth was significantly higher in the non-EDA group than in the EDA group (*P* = 0.042, *d* = 0.234). Finally, women who smoked were not included.

**Table 1:** Characteristics of comparison groups for all participants, with and without EDA and with and without synOT.

	All (N = 83)		non-EDA (n = 31)		EDA (n = 52)		*P*	*d*	non-synOT (n = 43)		synOT (n = 40)		*P*	*d*
	*n*	*M* (*SD*)	*n*	*M* (*SD*)	*n*	*M* (*SD*)			*n*	*M* (*SD*)	*n*	*M* (*SD*)		
Age (y)	83	33.5(3.7)	31	33.4 (4.0)	52	33.6(3.6)	.364		43	33.6 (4.1)	40	33.4 (3.4)	.398	
Gestational age	83	39.6 (1.0)	31	39.8 (0.8)	52	39.6 (1.1)	.381		43	39.6 (1.0)	40	39.7 (1.0)	.845	
Maternal weight (kg)	83	61.1 (7.5)	31	62.8 (8.8)	52	60.1 (6.5)	.120		43	61.6 (8.2)	40	60.5 (6.7)	.518	
Married (n, %)	82	98.8%	31	100.0%	51	98.1%	1.000		43	100.0%	39	97.5%	.482	
Work (n, %)	72	86.8%	25	80.6%	47	90.4%	.315		36	83.7%	36	90.0%	.523	
Educational > 15 year (n, %)	82	98.8%	30	96.8%	52	100.0%	.373		43	100.0%	39	97.5%	.482	
PCOS (n, %)	8	9.6%	2	6.5%	6	11.5%	.704		5	11.6%	3	7.5%	.714	
Intention to breastfeed < 13 weeks (n, %)	48	57.8%	19	61.3%	29	55.8%	.653		26	60.5%	22	55.0%	.661	
Blood loss during parturition (g)	83	574 (300)	31	586 (345)	52	567 (272)	.759	.070	43	548 (282)	40	603 (317)	.400	.186
Hb, early postpartum (mg/dL)	83	9.8 (1.3)	31	10.0 (1.5)	52	9.7 (1.3)	.433	.179	43	10.2 (1.3)	40	9.5 (1.3)	**.012**	**.567**
Baby weight (g)	83	3020 (309)	31	3067 (301)	52	2994 (313)	.298	.238	43	2998 (306)	40	3045 (313)	.494	.149
SSC within 1 h after childbirth† (n, %)	76	91.6%	31	100.0%	45	86.5%	**.042**	**.234**	40	93.0%	36	90.0%	.706	.054

*M* = Mean, *SD* = Standard deviation, *P* = .05 level, d = Cohen's d/Cramer's V, PCOS = polycystic ovarian syndrome, IU = international unit, mU = mili-unit, † = SSC within 1 h after childbirth or SSC 1 h after childbirth included not-STS (n = 4). Significant values are in bold.

Women with combined spinal epidural anesthesia accounted for 38.5% (n = 20) in the EDA group. The median and IQR dose of fentanyl in the EDA group (n = 52) was 121 µg (range, 77–192 µg). The median and IQR dose of intrapartum synOT in the synOT group (n = 40) was 689 mU (range, 272–2820 mU).

### Breast milk supply

At 4 months postpartum, 89.2% were still breastfeeding (Table 2). None of the reasons given for interrupting breastfeeding was due to illness or work. Lactation recording was started at an average of 3.1 days (SD 0.2) at early postpartum (T1) and at an average of 31.6 days (SD 1.8) at 1 month postpartum (T2).

**Table 2:** Medical interventions, breastfeeding conditions, and maternal salivary oxytocin levels.

	All (N = 83)		Non-EDA (n = 31)		EDA (n = 52)		*P*	*d*	Non-synOT (n = 43)		synOT (n = 40)		*P*	*d*
	*n*	*M* (*SD*)	*n*	*M* (*SD*)	*n*	*M* (*SD*)			*n*	*M* (*SD*)	*n*	*M* (*SD*)		
Fentanyl dose (µg)	83	93 (110)	31	0.0 (0.0)	52	148 (106)	–	–	43	52.6(77.0)	40	136 (124)	**< .001**	**.815**
Intrapartum SynOT (mU)	83	873 (1974)	31	325 (958)	52	1200 (2331)	**.020**	**.451**	43	0.0 (0.0)	40	1812 (2539)	–	–
Total SynOT (IU)	83	10.7 (6.8)	31	10.3(7.2)	52	10.9 (6.6)	.698	.890	43	9.2 (6.1)	40	12.3 (7.2)	**.030**	**.473**
Salivary oxytocin level	83	8.7 (3.9)	31	9.7 (4.3)	52	8.0 (3.5)	**.055**	**.442**	43	9.2 (4.1)	40	8.1 (3.6)	.224	.269
Continued breastfeeding														
1-month	82	98.8%	30	96.8%	52	100.0%	1.000	.085	43	100.0%	39	97.5%	.482	.114
4-month	74	89.2%	26	83.8%	45	86.5%	.144	.189	40	93.0%	31	77.5%	.081	.206
Date of commencement of measurement (days)														
Early postpartum	83	3.1 (0.2)	31	3.1 (0.3)	52	3.0 (0.2)	.597	.120	43	3.0 (0.2)	40	3.1 (0.3)	.287	.240
1-month	83	31.6 (1.8)	31	31.6 (1.7)	52	31.6 (1.8)	.995	.010	43	31.5 (1.8)	40	31.8 (1.8)	.433	.173
Breast milk supply (g)														
Early postpartum	83	174 (133)	31	216(121)	52	149(135)	**.025**	**.520**	43	186(131)	40	161(136)	.383	.193
1-month	83	530 (277)	31	612(216)	52	481(300)	**.036**	**.483**	43	501(249)	40	561(305)	.329	.216
Breastfeeding frequency†														
Early postpartum	83	10.4 (5.1)	31	11.9 (5.8)	52	9.4 ( 4.4)	**.032**	**.495**	43	9.9 (4.7)	40	10.8 (5.4)	.427	.175
1-month	83	9.2 (3.8)	31	9.6 (2.4)	52	9.0 (4.4)	.431	.157	43	9.5 (3.2)	40	9.0 (4.4)	.502	.148
Breastfeeding total hours (minutes)‡														
Early postpartum	83	229 (117)	31	251 (134)	52	216 (104)	.192	.299	43	228 (125)	40	230 (108)	.937	.018
1-month	83	188 (87)	31	203 (65)	52	179 (98)	.190	.273	43	200 (91)	40	176 (82)	.204	.282
The initiation of lactogenesis stage II	83	90.9 (24.2)	31	92.1 (28.7)	52	90.2 (21.3)	.728	.079	43	91.9 (27.3)	40	89.9 (20.6)	.708	.083
High breastfeeding (as greater than 80% in total infant diet)														
1-month	41	49.4%	20	64.5%	21	40.4%	**.042**	**.233**	21	51.2%	20	48.8%	1.000	.112
4-month	52	62.7%	24	77.4%	28	53.8%	**.037**	**.236**	27	51.9%	25	48.1%	1.000	.003

*M* = Mean, *SD* = Standard deviation, *P* = .05 level, *d* = Cohen's d or Cramer's V, IU = international unit, mU = mili-unit, † = included zero frequency (excluded not-direct breastfeeding and pumping stimulation), ‡ = included zero minutes (Excluded not-direct breastfeeding and pumping stimulation). Significant values are in bold.

Milk supply averaged 174 g (SD 133; Min 0, Max 540) at early postpartum and 530 g (SD 277; Min 0, Max 1336) at 1 month postpartum. At early postpartum, the mean milk supply was significantly lower in the EDA group [149 g (SD 135)] than in the non-EDA group [216 g (SD 121)] (*P* = 0.025, *d* = 0.520). Even at 1 month postpartum, the mean milk supply was significantly lower in the EDA group [481 g (SD 300)] than in the non-EDA group [612 g (SD 216)] (*P* = 0.036, d = 0.483). There was no significant difference in milk supply with or without synOT until the second stage of parturition.

Breastfeeding frequency in early postpartum was significantly lower in the EDA group [9.4 times (SD 4.4)] than in the non-EDA group [11.9 times (SD 5.8)] (*P* = 0.032, *d* = 0.495). There were no significant differences in the breastfeeding frequency at 1 month postpartum between the EDA group and the non-EDA group, and between *with synOT* and *without synOT*, as well as in the initiation of lactogenesis stage II.

In terms of breastfeeding rates, at 1 month postpartum, the High breastfeeding (> 80% of total infant diet) rate was significantly lower in the EDA group (40.4%) than in the non-EDA group (64.5%) (*P* = 0.042, *V* = 0.233). At 4 months postpartum, the High breastfeeding rate was significantly lower in the EDA group (53.8%) than in the non-EDA group (77.4%) (*P* = 0.037, *V* = 0.236).

Table 3 shows the Pearson correlation coefficients among the independent variables, salivary OT, and breast milk supply. In Table 3, age showed a weak negative correlation (*r* = 0.223, *P* = 0.043), and breastfeeding frequency at early postpartum and 1 month postpartum showed a positive correlation with 1 month postpartum breast milk supply (g) (*r* = 0.306, *P* = 0.005 and *r* = 0.331, *P* = 0.002, respectively). Moreover, early postpartum milk supply was strongly correlated with 1 month postpartum breast milk supply (*r* = 0.684, *P* < 0.000).

**Table 3:** Pearson correlation coefficients between independent variables and salivary oxytocin, breast milk supply.

	Salivary oxytocin		Early postpartum breast milk supply		1-month postpartum breast milk supply	
	*r*	*P*	*r*	*P*	*r*	*P*
Age (y)	− .086	.439	− **.267**	**.015**	− **.223**	**.043**
Baby weight (g)	− .146	.189	.118	.290	**.234**	**.034**
Fentanyl dose (µg)	− **.224**	**.042**	.078	.484	.015	.896
Intrapartum SynOT (mU)	− .104	.351	.080	.471	**.271**	**.013**
The initiation of lactogenesis stage II	− .037	.743	− **.329**	**.002**	− .206	.061
Breastfeeding frequency†						
Early postpartum	− .002	.987	**.248**	**.024**	**.306**	**.005**
1-month	− .002	.987	**.347**	**.001**	**.331**	**.002**
Breastfeeding total hours (minutes)‡						
Early postpartum	− .025	.820	.146	.186	.198	.072
1-month	.173	.120	.121	.280	**.259**	**.019**

*r* = Pearson correlation, *P* = .05 level, mU = mili-unit, † = included zero frequency (excluded not-direct breastfeeding and pumping stimulation), ‡ = included zero minutes (excluded not-direct breastfeeding and pumping stimulation). Significant values are in bold.

### Salivary oxytocin levels

Table 2 shows that the mean salivary OT level at 8.7 pg/mL (SD 3.9). The salivary OT levels were 8.0 pg/mL (SD 3.5) in the EDA group and 9.7 pg/mL (SD 4.3) in the non-EDA group, showing a lower level in the EDA group (*P* = 0.055,* d* = 0.442). In Table 3, the salivary OT level showed a weak negative correlation (*r* = 0.224, *P* = 0.042) with the Fentanyl dose (µg).

Subgroup analysis in the EDA group showed that the salivary OT level was 7.06 pg/mL (SD 3.58, n = 19) in the fentanyl high-volume group (> 150 µg) and 8.60 pg/mL (SD 3.44, n = 33) in the medium-volume group, with a trend towards a lower salivary OT level in the high-volume group (*P* = 0.065, *d* = 0.443).

## Discussion

This study examined the effects of intrapartum synOT and EDA on breast milk supply (g), breastfeeding rates, and maternal salivary OT levels. Women who delivered using EDA had a lower milk supply in early postpartum and 1 month postpartum and a lower breastfeeding rate at 4 months postpartum than women who delivered without using EDA. These results suggest that EDA may reduce the early postnatal salivary OT level and long-term breast milk supply of mothers.

Our findings align with other studies showing that EDA reduces breastfeeding rates^10,31^ and endogenous OT levels^32^. Decreased endogenous OT levels are thought to *weaken* the primitive reflexes of the newborn, *reduce* sucking, and consequently *decrease* the milk supply^33–35^. The significantly lower frequency of SSC within 1 h in the EDA group could also be a possible reason for the reduced milk supply. Breastfeeding should be started immediately or as soon as possible after delivery. Sensitivity analyses have shown that women who performed SSC breastfeed for longer periods^36^. In a previous study, women randomly administered fentanyl above 150 µg reported reduced primitive reflexes and considerable feeding difficulties in their infants at 6 weeks of age^37^. Subgroup analyses in this study revealed that the salivary OT level tended to be lower in the high-dose group with fentanyl > 150 µg than in the medium-dose group, in support of previous studies.

In Japan, there is a small number of people who choose epidural deliveries, but this number has recently been increasing. The effects of EDA on breastfeeding are not well known and remain inadequately reflected in breastfeeding care. In the present study, early postpartum milk supply was found to be strongly correlated with 1 month postpartum breast milk supply. Thus, it is essential for the clinical staff to be aware of these effects and provide early support during hospitalization, particularly as the present study suggested that EDA has a long-term impact on breastfeeding. From the time of pregnancy, it is advisable to provide women with information indicating that the administration of an epidural may affect the release of endogenous oxytocin, potentially influencing the let-down reflex and mother-infant bonding. All infants should be placed in SSC with the mother immediately after birth, unless medically contraindicated^36^. During the hospital stay, 24-h rooming-in promotes the establishment of breastfeeding and the transition to motherhood^38^. Frequent breastfeeding may supplement the potential for endogenous OT release in the early postpartum period. In fact, it was shown in the present study that breastfeeding frequency has a positive correlation with breast milk supply. Moreover, assistance may be required in positioning for breastfeeding owing to medical interventions such as IV treatment or the use of urinary catheters. Taken together, it is important to remember that if an epidural is necessary or preferable for pain relief during labor, it should not be relinquished because of concerns about potential breastfeeding problems. Choosing an epidural during labor does not preclude breastfeeding. It is important for women to adequately learn and receive information about epidurals to make an informed decision. A more comprehensive breastfeeding care is therefore needed for women who choose EDA delivery. To our knowledge, this is the first study to observe the actual changes in milk supply among women who use EDA during delivery. As EDA deliveries have been increasing rapidly, the results of the present study are anticipated to contribute to the future development of a more optimal breastfeeding care.

In the present study, the comparison of the breastfeeding rates between the 2 groups in terms intrapartum synOT showed no significant difference. This outcome differed from the initially assumed result, but supported the finding of a study that found no significant difference in the complete breastfeeding rates at 3 and 6 months postpartum with intrapartum synOT^39^. Intrapartum synOT has been shown to predict early breastfeeding inhibition, but its long-term effects have not been found, suggesting that the impact of intrapartum synOT is likely to be limited and dose-dependent^40,41^. One negative effect of synOT during parturition is the reduction of the expression of primitive reflexes associated with breastfeeding^17,42^ However, the intrapartum synOT dose in the present study was very low, and in fact was significantly less than half of the doses of previous studies. It has also been suggested that the effects of intrapartum synOT may be dose-dependent^43^, and that a low-dose regimen may have no effects on breastfeeding-related indicators. Cochrane review results of high-dose versus low-dose OT infusion regimens have revealed no significant differences in the rates of vaginal delivery not achieved within 24 h or of cesarean section^4^. A recent multicenter study observed that participants receiving low doses of OT had a higher rate of successful inductions and a lower rate of cesarean sections than participants receiving high doses of OT^3^. The present study suggests that using synOT at low doses during labor does not significantly affect breastfeeding.

### Strengths and limitations

The strength of this study was that it identified the specific 24-h milk supply by test weights in a standardized manner. Although test weights (i.e., weighing an infant before and after breastfeeding to assess milk intake) remain controversial, they are commonly used methods in studies of intake in normal neonates, with some studies showing 85% of test weights within 5 mL error^44^. Additionally, a 24-h measurement will be more accurate because the change in an infant’s test weight over 24 h is greater than the change in an infant’s test weight after a single feeding^45,46^.

On the other hand, those who did not continue breastfeeding may have dropped out. This possibility was anticipated in advance by setting the study’s eligibility criteria to include those who planned to continue breastfeeding for at least 12 weeks postpartum (including mixed feeding). Also during recruitment, it was explained in plain language that the study’s main purpose was to make observations, and a commitment was made to respect the breastfeeding style of the subjects.

## Conclusions

Women who delivered using EDA had a lower milk supply in the early postpartum period and lower breastfeeding rates at 4 months postpartum than women who delivered without using EDA. Intrapartum synOT had no significant effect on breastfeeding rates. Thus, women who deliver using EDA need support for increased milk supply in the early postpartum period.

## Data Availability

All data generated or analyzed during this study are included in this published article.

## Abbreviations

synOT: Synthetic oxytocin
EDA: Epidural anesthesia
